# Molecular Detection and Genetic Characterization of Bovine Kobuvirus (BKV) in Diarrhoeic Calves in a Central Italy Herd

**DOI:** 10.1155/2023/6637801

**Published:** 2023-05-13

**Authors:** Cecilia Righi, Valentina Curini, Claudia Torresi, Cesare Cammà, Silvia Pirani, Valeria Di Lollo, Paola Gobbi, Monica Giammarioli, Giulio Viola, Michela Pela, Francesco Feliziani, Stefano Petrini

**Affiliations:** ^1^Istituto Zooprofilattico Sperimentale Umbria-Marche, “Togo Rosati“, Perugia 06126, Italy; ^2^Istituto Zooprofilattico Sperimentale dell'Abruzzo e del Molise G. Caporale, Teramo 64100, Italy; ^3^Veterinary Practitioner, San Ginesio 62026, Italy

## Abstract

Bovine kobuvirus (BKV) is an infectious agent associated with neonatal calf diarrhoea (NCD), causing important economic losses to dairy and beef cattle herds worldwide. Here, we present the detection rate and characterize the genome of BKV isolated from diarrhoeic calves from a Central Italy herd. From January to December 2021, we collected blood samples and nasal and rectal swabs from 66 calves with severe NCD between 3 and 20 days of age. After virological (bovine coronavirus, bovine viral diarrhoea virus, and bovine rotavirus), bacteriological (*Escherichia coli* spp. and *Salmonella* spp.), and parasitological (*Cryptosporidium* spp., *Eimeria* spp., and *Giardia duodenalis*) investigations, we detected BKV using the metagenomic analysis. This result was confirmed using a specific polymerase chain reaction assay that revealed the number of BKV-positive nasal (24.2%) and rectal swabs (31.8%). The prevalence of BKV was higher than that of BCoV. Coinfection with BKV and BCoV was detected in 7.5% of the rectal swabs, highlighting the involvement of another infectious agent in NCD. Using next generation sequencing (NGS) approach, it was possible to obtain the complete sequence of the BKV genome from other two rectal swabs previously analysed by real-time PCR. This is the first report describing the whole genome sequence (WGS) of BKV from Italy. The Italian BKV genomes showed the highest nucleotide sequence identity with BKV KY407744.1, identified in Egypt in 2014. The sequence encoding VP1 best matched that of BKV KY024562, identified in Scotland in 2013. Considering the small number of BKV WGSs available in public databases, further studies are urgently required to assess the whole genome constellation of circulating BKV strains. Furthermore, pathogenicity studies should be conducted by inoculating calves with either only BKV or a combination with other enteric pathogens for understanding the probable role of BKV in NCD.

## 1. Introduction

Bovine kobuvirus (BKV) is a member of the Picornaviridae family, genus *Kobuvirus*, species *Aichivirus B* [1, 2].

Kobuvirus-like infections play an important role in animal and human health [3].

The BKV ssRNA genome encodes a single large polyprotein (L) that is cleaved into three structural (VP0, VP3, and VP1) and seven nonstructural (2A–2C and 3A–3D) proteins [4]. VP1 is the most variable immune determinant protein, and thus, is suitable for genotyping [1]. Conversely, the sequence of the 3D region is highly conserved and encodes the enzyme RNA-dependent RNA polymerase.

The BKV is one of the factors contributing to the clinical manifestations of neonatal calf diarrhoea (NCD) affecting calves younger than one month [5]. Transmission occurs via direct contact with infected animals via the faecal-oral route [6], and a multihost landscape has been revealed in terms of host order, family, and species, suggesting that wildlife may represent an extensive reservoir of BKV for livestock animals [7]. Moreover, BKV-associated NCD results in substantial economic loss to dairy and beef cattle producers owing to growth disorders, cost of treatment, and death of sick animals [8].

BKV was isolated for the first time in 2003 in Japan in a Vero cell culture medium of HeLa cells containing 10% calf serum, and subsequently, from faecal samples from calves 2–35 days old [9]. Thereafter, its presence has been reported in different countries such as Japan [9], Thailand [10], South Korea [11], China [12], Hungary [13], Belgium [14], the Netherlands [15], Italy [7, 16], the United Kingdom [17], Turkey [17], Egypt [18], Brazil [19], Bangladesh [20], Vietnam [20], Canada [21], and the USA [22], suggesting a global widespread.

BKV has been recognised globally for approximately two decades, and morbidity and mortality are still elevated in different regions [8]. To date, information on its detection in Italy is very limited [16], and its role in the pathogenesis of diarrhoea in calves is not fully understood. The present study aimed to fill this gap in knowledge by presenting concrete information on the prevalence and characterising of the genome of BKV isolated from diarrhoeic calves in Central Italy.

## 2. Materials and Methods

A herd in Central Italy (Marche region) comprising approximately 120 Friesian cattle was monitored for one year, during which 66 calves out of a total 85 between 3 and 20 days old, showed severe diarrhoea. All 66 calves showed dehydration, loss of appetite, anorexia, and sensory depression culminating in death, which was not prevented by rehydration, nutrient, and antibiotic therapies.

Due to the compelling clinical picture of these cases, further investigations were conducted.

The anamnesis indicated that the animals were treated with antiparasitic drugs every 6 months and regularly vaccinated against bovine alphaherpesvirus 1 (BoHV-1), bovine respiratory syncytial virus (BRSV), bovine parainfluenza-3 (BPI-3) virus, bovine viral diarrhoea virus (BVDV), bovine coronavirus (BCoV), bovine rotavirus (BRV), and *Escherichia coli.*

Between January 2021 and December 2021, using a convenience sampling strategy, we collected blood samples and nasal and rectal swabs for virological investigations (BCoV and BVDV) from each of the 66 sick animals.

Nasal and faecal suspensions were prepared by diluting the swabs in 3 ml transport fluid composed of minimum essential medium (MEM). Using the same extraction protocol, the nucleic acids were isolated from these samples using the QIAamp Viral RNA Mini Kit–Spin Protocol (QIAGEN viral kit, Hilden, Germany), according to the manufacturer's instructions.

BCoV was detected using the SuperScript™ III one-step real-time polymerase chain reaction (PCR) system with the Platinum™ Taq DNA Polymerase (Thermo Fisher, Superscript, California, USA) [23]. Thermal cycling conditions were as follows: 60°C for 15 min, 95°C for 2 min, and 95°C for 15 s, followed by 45 cycles at 95°C for 15 s, 55°C for 30 s, and 60°C for 1 min. For BVDV detection, the TaqMan Fast Virus 1-Step Master Mix (4×) (Thermo Fisher) was used [24, 25]. The primers and probes used for real-time PCR of different viruses are listed in Table 1.

In addition, to rule out other differential diagnoses, a stool sample was taken from each calf for bacteriological (*E. coli* spp. and *Salmonella* spp.) and parasitological (*Cryptosporidium* spp., *Eimeria* spp., and *Giardia duodenalis*) investigations. Moreover, BRV infection was investigated using an Ag-ELISA test (IDEXX Laboratories, Inc. One IDEXX Drive Westbrook, Maine, USA).

Bacteriological investigations were conducted using the protocols previously described by Carter and Cole [26]. Parasitological investigations were conducted as described by Cringoli et al. [27].

One positive BCoV rectal swab sample (sample 1, 2021.TE.300917.1.1) was processed by next generation sequencing to identify other pathogens eventually involved in NCD.

After Turbo DNAse (Thermo Fisher Scientific, Waltham, MA, USA) incubation, and RNA purification by RNA Clean & Concentrator™-5 Kit (Zymo Research, Irvine, CA, USA), purified RNA was processed using a modified sequence-independent single-primer-amplification (SISPA) method to obtain dsDNA [28, 29].

The library was processed using Illumina DNA Prep kit (Illumina Inc., San Diego, CA, USA) and sequenced using NextSeq 500/550 Mid Output Reagent Cartridge v2, 300 cycles, and standard 150 bp paired end reads. After evaluating the quality and trimming the raw reads data using FastQC v0.11.5 and Trimmomatic v0.36 (Germany) [30], respectively, NGS analysis was performed by the software platform CZ IDseq (v4.0) (https://czid.org/) which identifies pathogens in metagenomic sequencing data. One of the most prevalent pathogens in the sample was BKV. Therefore, a mapping of the reads assigned to BKV vs. best reference (KY407744) was performed by iVar (v1.3.1) (intrahost Variant analysis of replicates; github.com/andersen-lab/ivar) to obtain consensus sequence.

To confirm the presence of BKV in stool, nasal, and rectal swab samples, a real-time PCR assay was performed using primers that targeted the 3D gene of BKV (Table 1) [21]. Thermal cycling conditions for BKV were as follows: 1 cycle at 50°C for 5 min, 1 cycle at 95°C for 20 s, followed by 45 cycles at 95°C for 3 s, and at 60°C for 30 s.

Two additional BKV-positive rectal swabs samples (sample 2, 2022.TE.15125.1.1 and sample 3, 2022.TE.15125.1.2) were sequenced to compare whole BKV genomes.

Finally, phylogenetic analysis by MEGA11 software [31] was performed using the three BKV genomes from this study and 14 complete genome sequences publicly available in NCBI (https://www.ncbi.nlm.nih.gov/nucleotide/). Moreover, a further phylogenetic analysis was carried out using 44 VP1 gene sequences available in NCBI and the VP1 gene sequences related to the three BKV genomes.

## 3. Results and Discussion

No pathogens were detected in the blood samples. In contrast, 15.15% (10/66) of nasal swabs and 7.57% (5/66) of rectal swabs were positive for BCoV.

BCoV detection in diarrhoeic calves of BCoV-vaccinated dams probably indicates that passive immunity did not protect the calves from infection by the wild-type BCoV strains during the first weeks of life.

Neither BVDV nor BRV were detected. No important pathogens were detected in bacteriological and parasitological investigations.

One BCov-positive sample was analysed by NGS. The taxonomical classification was performed after quality filtering and depleting the host sequences from the metagenomic sequencing data of sample 1 using CZ IDseq software. In addition to bacteria common in human gut flora, such as Phocaeicola, Faecalibacterium, Bacteroides, and Prevotella, the software assigned 48,453 and 12,955 reads to BCoV and BKV, respectively. Mapping vs. BKV best reference (KY407744) produced a complete consensus sequence of 8,301 bp with an average vertical coverage (Vcov) of 108× and a horizontal coverage (Hcov) of 90.41% (GenBank accession number: OP805596).

Real-time PCR confirmed the metagenomics results. In particular, 24.24% of nasal swabs (16/66) were positive for BKV. Furthermore, 31.82% (21/66) of rectal swabs were positive for BKV and 7.57% (5/66) for BCoV. Moreover, coinfection with BKV and BCoV was detected in 7.57% (5/66) of the rectal swabs (Table 2).

From samples 2 and 3, 1,270,996 and 30,172 reads mapped to BKV, respectively. Mapping against KY407744 produced complete consensus sequences for both samples (sample 2 : 8289 bp with Vcov 5475× and Hcov 90.27% (GenBank accession number: OP805597); sample 3 : 8281 bp with Vcov 360× and Hcov 90.25% (GenBank accession number: OP805598)).

**Figure 1:**
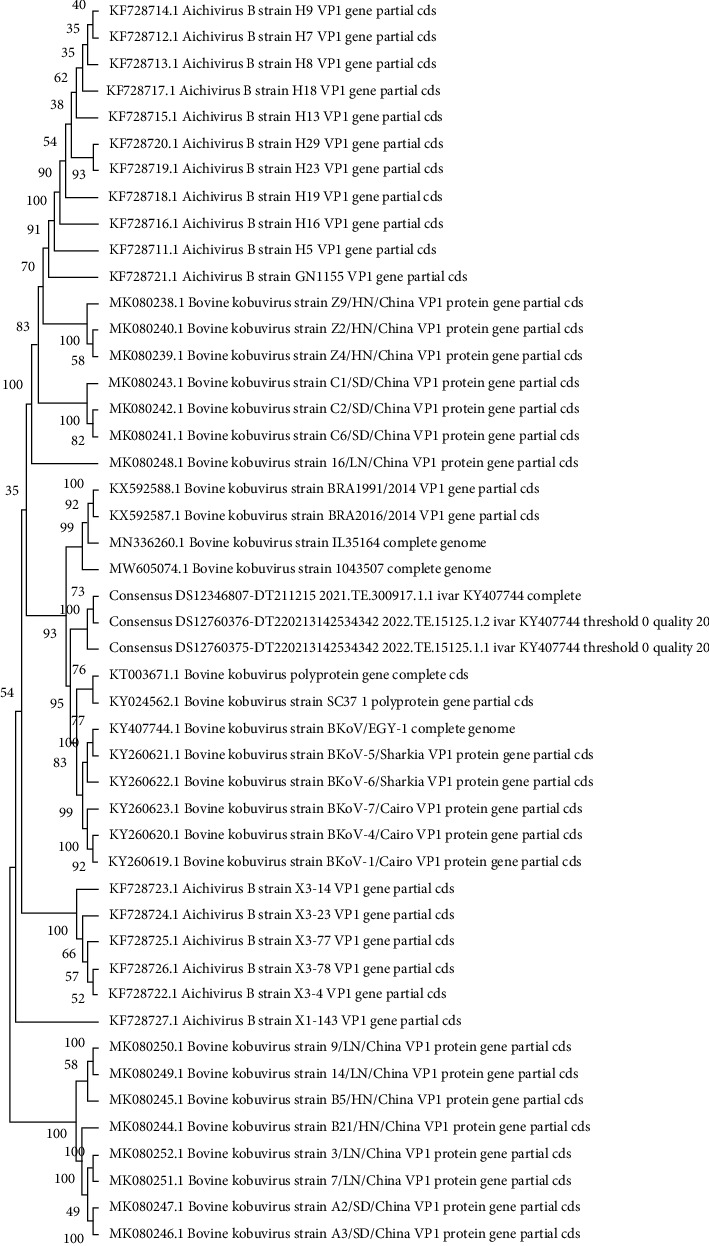
Phylogenetic tree analysis of complete BKV genomes was performed using the MEGA software package version 1. The evolutionary history was inferred using the maximum likelihood method and general time-reversible model, with bootstrap analysis (100 replicates). Consensus_DS12346807-DT211215_2021.TE.300917.1.1 = sample 1_OP805596; consensus_DS12760375-DT220213142534342_2022.TE.15125.1.1 = sample 2_OP805597; and consensus_DS12760376-DT220213142534342_2022.TE.15125.1.2 = sample 3_OP805598.

Phylogenetic analysis of the three consensus sequences obtained in this study and the complete sequences publicly available revealed that the three samples clustered together, confirming that the genome sequences were strictly correlated. Moreover, they displayed a high nucleotide (nt) identity (90.41%, 90.27%, and 90.25%) with the Egyptian sequence (BKV KY407744) identified in 2014 (Figure 1).

Sequences analysis showed that the three BKV sequences had a genome organization similar to that of other BKVs from GenBank, including an open reading frame (from 754 nt to 8145 nt for samples 1 and 2 and from 755 nt to 8,146 nt for sample 3) that encoded 2,463 amino acids. The three amino acid sequences were 100% identical to the Egyptian BKV strain polyprotein.

**Figure 2:**
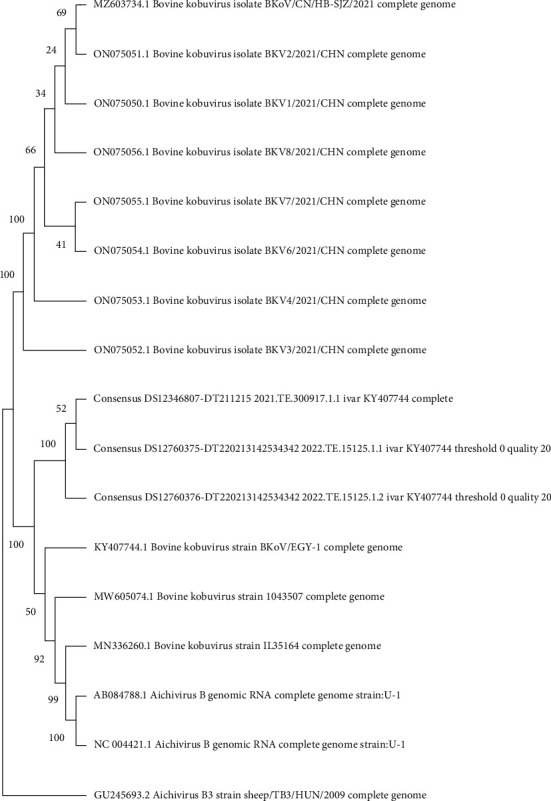
Phylogenetic tree analysis of the BKV VP1 gene sequences was performed using the MEGA software package version 1. The evolutionary history was inferred using the maximum likelihood method and a Tamura-Nei model, with bootstrap analysis (100 replicates).

As the number of complete BKV genomes available in NCBI is very limited, phylogenetic analysis was conducted based on the VP1 gene sequences in the NCBI nucleotide database. The analysis displayed the highest nt identity with BKV KY024562 detected in Scotland in 2013 (88.76% nt identity) (Figure 2).

BKV was detected in diarrhoeic and asymptomatic cattle in several countries on four continents [1]. This virus was also reported in Italy in both domestic and wild ungulates [7, 16].

Although the virus causes considerable economic losses to cattle producers, it has never been thoroughly investigated, probably because it has not been included on the list of agents causing notifiable diseases and is not a zoonotic pathogen.

In this study, using specific real-time PCR, we confirmed the significant involvement of BKV in NCD in 77.65% as the attack risk of calf diarrhoea in this herd. This pathology is frequently caused by a combination of predisposing factors such as age, unfavourable environmental conditions, an inefficient immune status, and infection with various other pathogens, including viral, bacterial, and protozoan agents [8, 21].

Interestingly, in the herd herein studied, we found evidence of coinfection with BKV and BCoV, which was also frequently reported in other articles [1, 8, 21]. Indeed, the animals, as nonprotective passive immunity against BCoV acquired, they were infected with the wild-type virus.

Moreover, in some cases, coinfection with BKV and BCoV worked synergistically at the enteric level. In the first phase, the calves had severe diarrhoea, which did not improve by rehydration, nutrient, and antibiotic therapies. Subsequently, the probably immunocompromised animals showed secondary complications (bacterial antibiotic resistance, hydroelectrolyte imbalances, acidosis, and hypoglycaemia) from which they ultimately died.

However, whether BKV plays a leading or contributing role in NCD remains to be clarified. We can hypothesise that it has played a role in viral superinfection in calves, causing their death.

In this study, we presented the whole BKV genomes from Italy for the first time. The limited availability of complete genomes has restricted the possibility of studying BKV evolution. Indeed, only 14 complete genome sequences of BKV are available in the NCBI database. Therefore, there is a compelling need for further studies assessing the whole genome constellation of circulating BKV strains.

Moreover, viral isolation and animal challenge studies are required to unveil the pathogenicity and the role of BKV in diarrhoea in calves [21].

Currently, there are no available commercial vaccines against BKV. Consequently, biosecurity measures within the farm are recommended to minimise the effects of BKV circulation among calves.

## Figures and Tables

**Table 1:** Primer pair candidates and probes used for detecting different viruses using real-time PCR.

Forward primer	5′-CAAATCTCGYCGCTCATTGTTC-3′	Amplicon of 114 bp	BKV
Reverse primer	5′-GTTGAGTAGAAGTAATCTGGGTTCT-3′		
Probe	5′-JUN-TCCCTACATCCGAACTCCAGGCT-QSY-3′		

Forward primer	5′-CTGGAAGTTGGTGGAGTT-3′	Amplicon of 85 bp	BCoV
Reverse primer	5′-ATTATCGGCCTAACATACATC-3′		
Probe	5′-FAM-CCTTCATATCTATACACATCAAGTTGTT-BHQ-3′		

Forward primer	5′-GRA-GTC-GTC-ART-GGT-TCG-AG-3′	Amplicon of 208 bp	BVDV
Reverse primer	5′-TCA-ACT-CCA-TGT-GCC-ATG-TAC-3′		
Probe	5′-FAM-TGC-YAY-GTG-GAC-GAG-GGC-ATG-C-TAMRA-3′		

**Table 2:** Prevalence of viruses in clinical samples of diarrhoeic calves.

Virus	Ages of calves with clinical signs (days)	Clinical signs	*Real-time PCR*	
			Nasal swabs	Rectal swabs
BKV	3–20	61/66 (92.42%)^a,b,d,e,f^	16/66 (24.24%)	21/66 (31.82%)
BCoV	4–10	5/66 (7.57%)^a,b,c,e,f^	10/66 (15.15%)	5/66 (7.57%)
BKV + BCoV	3–20	5/66 (7.57%)^a,b,d,e,f^	0/66 (0%)	5/66 (7.57%)

BKV, Bovine kobuvirus; BCoV, Bovine coronavirus; ^a^Severe diarrhoea; ^b^dehydration and anorexia; ^c^respiratory symptoms; ^d^poor mobility; ^e^sensory depression; ^f^death.

## Data Availability

The authors confirm that the data supporting the findings of this study are available within the article, and the obtained sequences were deposited in GenBank.
